# Structural Characterisation and Mechanical FE Analysis of Conventional and M-Wire Ni-Ti Alloys Used in Endodontic Rotary Instruments

**DOI:** 10.1155/2014/976459

**Published:** 2014-01-20

**Authors:** Diogo Montalvão, Francisca Sena Alçada, Francisco Manuel Braz Fernandes, Sancho de Vilaverde-Correia

**Affiliations:** ^1^School of Engineering and Technology, University of Hertfordshire, College Lane, Hatfield, Hertfordshire AL10 9AB, UK; ^2^The Dental Implant and Gingival-Plastic Surgery Centre, 717 Christchurch Road, Bournemouth, Dorset BH7 6AF, UK; ^3^CENIMAT/I3N, Materials Science Department, Faculty of Science and Technology, New University of Lisbon, Campus of Caparica, 2829-516 Caparica, Portugal; ^4^Department of Endodontics, School of Dental Medicine, University of Pennsylvania, 3451 Walnut Street, Philadelphia, PA 19104, USA

## Abstract

The purpose of this study is to understand how the M-Wire alloy conditions the mechanical flexibility of endodontic rotary files at body temperature.Two different rotary instruments, a Profile GT 20/.06 and a Profile GT Series X 20/.06, were selected due to their geometrical similarity and their different constituent alloy. GT series X files are made from M-Wire, a Ni-Ti alloy allegedly having higher flexibility at body temperature. Both files were analysed by X-Ray Diffraction and Differential Scanning Calorimetry to investigate phase transformations and the effects of working temperature on these different alloys. Mechanical behaviour was assessed by means of static bending and torsional Finite Element simulations, taking into account the nonlinear superelastic behaviour of Ni-Ti materials. It was found that GT files present austenitic phase at body temperature, whereas GT series X present R-phase at temperatures under 40°C with a potential for larger flexibility. For the same load conditions, simulations showed that the slight geometrical differences between the two files do not introduce great disagreement in the instruments' mechanical response. It was confirmed that M-Wire increases the instrument's flexibility, mainly due to the presence of R-phase at body temperature.

## 1. Introduction

Stainless steel instruments are rigid and therefore unsuited for large apical enlargement in thin curved canals [1]. Ni-Ti alloys have superior properties in ductility, fatigue, recoverable strain, biocompatibility, and corrosion resistance [2]. Ni-Ti alloys' lower Young's modulus and superelastic behaviour are paramount for preparation of anatomically complex root canals, as flexibility preserves dental structure, limits apical transport, reduces the risk of iatrogenic mistakes, and ultimately allows for irrigants to flow deeper in canals, towards the apical constriction [3, 4]. Also, some studies claim that there is now evidence [5] that Ni-Ti instruments yield better clinical prognosis in endodontics when compared to their stainless steel counterparts considered alone.

However, Ni-Ti files present a higher risk of unnoticed fracture inside canals [6], contrary to stainless steel files that often present visible signs of plastic deformation [7]. Unexpected premature file fracture is of paramount concern as it might hinder clinical outcome. Some published studies show that Ni-Ti instruments fracture at a frequency ten times greater than their stainless steel counterparts [8, 9]. Furthermore, Ni-Ti fragments are up to seven times less likely to be removed from inside root canals, even by experienced endodontists [10]. This explains the current interest in the subject by several researchers [11–17].

Recent fabrication of superelastic files is mostly focused on geometrical details, with emphasis on cross-sectional design [18–21]. Alongside, there have been significant developments in material properties. Endodontic files Ni-Ti should ideally have excellent mechanical strength properties (measured by both the ultimate tensile strength and the fatigue strength), it should be flexible enough to avoid canal transportation, and its hardness should be large enough to allow for good cutting efficacy [22, 23].

Flexibility in Ni-Ti files due to their superelastic behaviour mainly depends on the crystallographic phases present in the alloy and the thermal, mechanical, and chemical treatments to which the alloy has been subjected. Ni-Ti alloys used in root canal treatment contain approximately 55%(wt) Ni and 45%(wt) Ti [22], equivalent to 50%(at) Ni and 50%(at) Ti.

Shape Memory Effect (SME) and Superelasticity (SE) are associated with the reversible nature of the martensitic transformation that happens in Shape Memory Alloys (SMA). This transformation may be thermally and/or mechanically induced. In the absence of applied stress, there are two temperatures, Ms and Mf, corresponding to the beginning and end of austenite (A) to martensite (M) transformation during cooling (direct transformation), and other two temperatures, As and Af, corresponding to the beginning and end of M to A transformation during heating (reverse transformation). Deformation of the thermally stable austenite (above Af) will lead to formation of stress induced martensite (SIM) above a critical stress that is temperature dependent. Due to crystallographic reversibility of this SIM transformation, the resulting strain (up to 10% in Ni-Ti alloys) may be completely recovered, which accounts for the superelastic effect. Such recoverable strain is much higher than the maximum elastic strain (up to 0.2%) that can be recovered in steels, namely, the stainless steel used in manual endodontic files. The A-M transformation may occur in one single step. However, according to the processing conditions [24–26], this A-M transformation may also go through an intermediate step [27, 28] (R-phase, first identified by Nagasawa in 1970 and later reported as a rhombohedral distortion of the parent A phase). R-phase has a much smaller stiffness than the other two phases, thus allowing for stress relaxation during deformation of endodontic instruments inside root canals; this means that the loads needed to deform files, either in bending or in torsion, are also much smaller. This characteristic, along with the very low thermal hysteresis of A-R-phase transformation (just a few degrees, compared to several tens of degrees for A-M), explains the great importance of determining the temperature range for R-phase existence in these endodontic files.

The crystallographic structure of superelastic Ni-Ti instruments' alloy at room temperature under no-load is austenite. Martensite in Ni-Ti alloys can be stress induced by a shear type of process, as it happens when rotary files are deformed by root canals' anatomical curvatures. Since austenite is the stable phase at this temperature, the material springs back to its original shape when stress is removed. Recent developments have improved the properties of rotary instrument files by using new alloys with special thermal treatments and new manufacturing processes [29]. The newly introduced to the market M-Wire is an example of such a Ni-Ti alloy nowadays seen in many files. According to its manufacturer, this alloy is thermomechanically processed in order to have a larger flexibility at body temperature than conventional Ni-Ti wire [30].

As Ni-Ti alloy properties depend on the materials' crystallographic phase, the present study will firstly characterize the material from two file types with geometrical similarities but different alloys: Profile GT 20/.06 (GT) and Profile GT Series X 20/.06 (GTX) files. Characterization will be carried out through X-Ray Diffraction (XRD) and Differential Scanning Calorimetry (DSC) as these are important methods to identify which phases are present and their evolution as a function of temperature [31, 32]. Secondly, some flexural and torsional simulations will be performed using Finite Element (FE) models, under the same loads and boundary conditions as those used by other authors [18–20].

The ultimate purpose is to investigate the presence of R-phase in M-Wire at body temperature and to understand how its presence interacts with endodontic file deformation and internal stresses relaxation. This is an all-encompassing study, following a smaller previous analysis [12], adding fundamental missing information regarding body temperature, which is a paramount parameter for NiTi instruments performance under clinical use.

## 2. Materials and Methods

### 2.1. Geometrical Considerations

**Figure 1:**
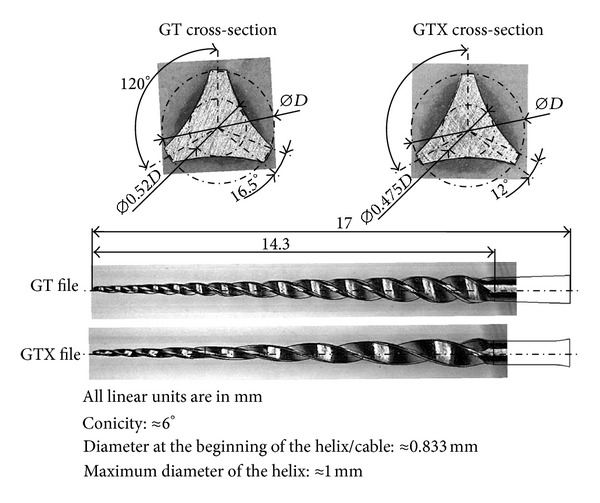
Cross-sectional and longitudinal geometries of the GT and GTX file instruments used in this study, where *D* stands for the local diameter.

Two as-received Ni-Ti rotary files with similar cross-sections—but different material properties—were selected for this study (Figure 1): Profile GT 20/.06 (GT) is made from conventional Ni-Ti alloy and GT series X 20/.06 (GTX) is made from M-Wire (both files were provided by Dentsply Tulsa Dental Specialties, Tulsa, OK). A Mitutoyo PJ-A300 with QM Data 200 profile projector, a Leica Zoom 2000 microscope, and a OPTIKA microscope with numeric ocular camera JEULIN 571205 were used to assist in the geometrical characterization of both GT and GTX files.

The purpose is that the CAD (Computer Assisted Design) geometrical models used in the FE analysis resemble the real files as much as possible, including variable radial land widths along the instruments' active parts. Usually, the file can be divided into three main parts: handle, shaft, and blade. The total length of both files compared in this study is 17 mm with a 14.3 mm long working part. In addition, both instruments have a 1 mm diameter at the transition section between the shaft and blade and approximately 6% taper. However, there are some differences between the GT and GTX files, which are shown in Figure 1. Compared with the GT file, the GTX has fewer spirals because of its larger pitch length. Besides, the pitch lengths of both the GT and GTX files are variable. Furthermore, the radial land widths on the GTX files are deeper than in the GT file, because of the narrower angled cutting edges. The CAD models, shown in Figure 1, accurately reproduce the actual dimensions of the working part of the instruments, which can be seen from the superposition of the models' edges over the photos, including different triple U-shaped cross-sections and variable land widths.

### 2.2. X-Ray Diffraction Tests

Six shafts of each file type—GT and GTX—were embedded in *epoxy* resin (*Epo-Thin, Buehler, Germany*) cylinder blocks and after cure they were slightly abraded with number 350 Silicon Carbide Waterproof Paper (Struers, Denmark). Shafts were then removed from their resin cast, their new flat surface was glued to a small piece of glass and flipped over before being included in a new epoxy resin cylinder. After cure, the metal was again abraded with sand paper until the shafts' maximum diameters were reached in order to obtain an area for analysis as large as possible. Samples were then removed from within the resin and mounted with silver colloid glue on the sample holder of the TTK-450 chamber, mounted on the XRD.

XRD measurements were performed using a diffractometer with a Bragg-Brentano mounting employing Cu-K*α* radiation (wavelength of 1.54 $\overset{´}{Å}$) produced by a rotating anode. The scanning was set up and programmed in the following way:

- test samples were analysed over a 2*θ* range from 30° to 55°, using an increment step size of 0.04°, with a counting time of 0.5 seconds at each step;
- angular scans were run for each file between temperatures of *T* (minimum) = −180°C and *T* (max) = 100°C (cooling and heating).

### 2.3. DSC Analysis

Transformation temperatures of A*⇔*R phases were determined by DSC. A shaft of each file was cut into five fragments, weighted, and analysed in a Setaram DSC92 calorimeter in the temperature range of −40°C to 100°C, with heating and cooling at 7.5°C/min.

### 2.4. Finite Element Modelling

GT and GTX files were modelled using Solidworks (three-dimensional computer-aided design software) and ANSYS 14.0 (computer-aided engineering software). Both geometries try to fit the real files as much as possible, with larger pitch length and variable radial land widths along the GTX instrument's active part. The geometrical model used in this section is improved in comparison to the one used in a previous simulation [12]: the concave (U-shaped flutes) cross-sections were actually assessed by cutting the files transversely as shown in Figure 1 and explained in Section 2.1 earlier. It may be seen that there are some differences between the GT and GTX cross-sections. This way, it is possible to also study the effects of the different geometries in the files' flexibility.

**Figure 2:**
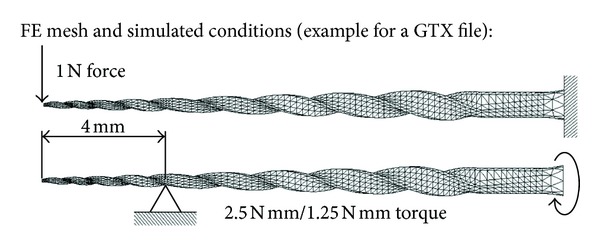
Finite Element model mesh with load and boundary conditions (example for a GTX file).

Tetrahedral structural solid elements (SOLID187), suited for complex geometries and for modelling irregular meshes [33], were used in the FE models. Meshes for the GT and GTX files consisted of 8681 nodes, 4441 elements and 4700 elements, 9119 nodes, respectively, with both being applied using mapped face meshing. The GTX file mesh is illustrated in Figure 2. The multikinematic hardening plastic material model was selected in the software to approximate the stress-strain relationships of the Ni-Ti alloys used in dentistry [18–20, 34]. For the general mechanical properties, a linear elastic isotropic material model was selected. The combination of both models for the material characterization was done following other works [18]. The files' material properties were modelled by considering the stress-strain relationships for conventional Ni-Ti and M-Wire found in the literature [34].

Two comparisons have been established between the GT and the GTX endodontic rotary files:

1. geometrical comparison: in this comparison both files were modelled considering the same material properties (conventional Ni-Ti), to evaluate the effect of the geometry alone on the instrument's performance;
2. material comparison: in this comparison the files were modelled using different stress-strain material models [35]; the Poisson's coefficient was considered 0.33 for both alloys [36].

The mechanical behaviour of the files was analysed by using similar loading and boundary conditions as in other works [18–20] (Figure 2):

1. evaluation of the equivalent (von Mises) stress distribution and tip deflection in bending: the instrument was clamped at the shaft's cable and subjected to a load of 1 N at its tip;
2. evaluation of the equivalent (von Mises) stress distribution and shaft rotation in torsion: the instrument was clamped 4 mm away from its tip and two different torques of 2.5 N mm and 1.25 N mm were applied at the edge of the shaft's cable, one at a time.

## 3. Results

### 3.1. X-Ray Diffraction Results

**Figure 3:**
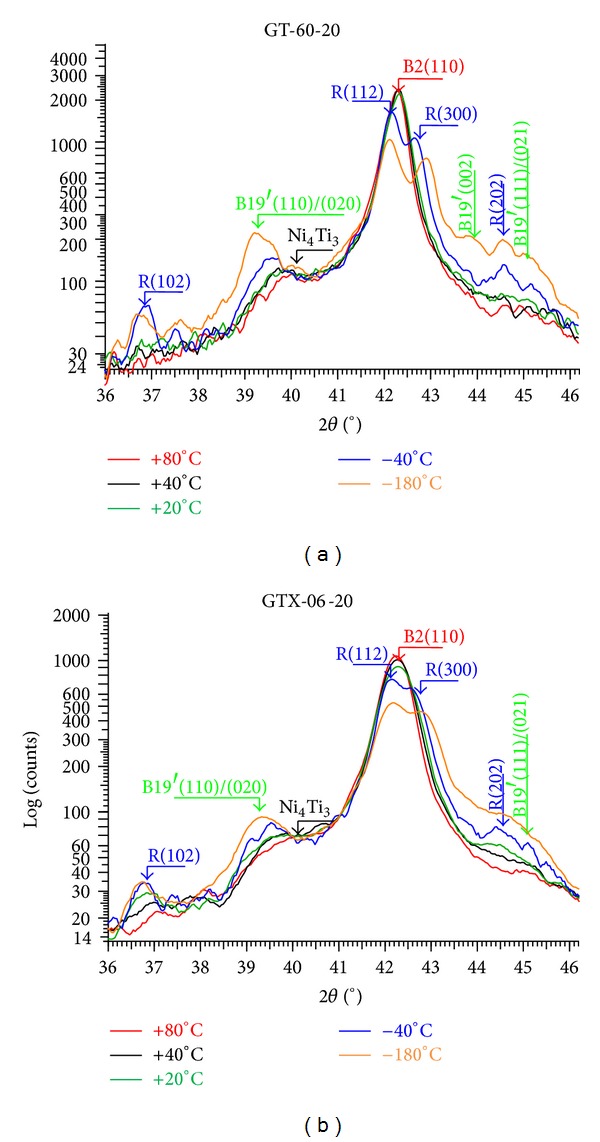
3D XRD patterns during cooling of both GT and GTX files, with identification of the austenite (B2), R-phase, and martensite (B19′) peaks: a selection of 5 scans at +80, +40, +20, −40, and −180°C, and a 3D view of this selected group of scans is shown.

A series of XRD scans, corresponding to different temperatures, were obtained during the cooling and heating cycles of both GT and GTX files' fragments. Only the patterns obtained during cooling are shown in Figure 3. *International Crystallographic Diffraction Data* (ICDD) cards were used to identify diffraction peaks of austenite—B2(110)-, R-phase—R(102), R(112), R(300), and R(202), and martensite—B19′.

In Figure 3 the diffraction peaks of both files are shown at five representative temperatures: 80, 40, 20, −40, and −180°C. At 80°C, both files are fully austenitic and at −180°C both are almost fully martensitic. R-phase presence appears as a broadening of the B2(110) because of the splitting of this peak into the two neighbouring R-phase diffraction peaks: R(112) and R(300).

A more detailed analysis of these results shows that:

- the austenite B2(110) peak's intensity decreases from 40°C downwards with a corresponding slight increase of the R-phase peaks' intensity for the GTX file while for the GT file this is more noticeable only below 20°C;
- R-phase peaks' intensity increases significantly during cooling, until −40°C is reached, in both files;
- martensite peaks' intensity increases more significantly for temperatures below −40°C, for both files;
- Ni_4_Ti_3_ precipitates are present in both files.

### 3.2. DSC Results

**Figure 4:**
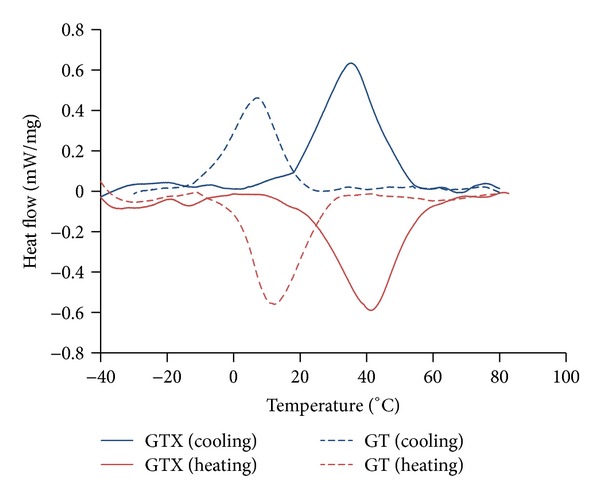
DSC curves for GT and GTX files obtained for cooling until −40°C.

Transformation temperatures were determined by DSC (Figure 4). The transformation temperatures of the GTX files are clearly shifted towards a higher temperature range and this result is consistent with the observations from XRD. Also from the XRD results we can say that, for the thermal cycle run between 80°C and −40°C, mostly the austenite*⇔*R-phase transformation was identified during DSC tests. In both files, from close to room temperature (20°C) up to sterilization temperature only the austenite*⇔*R-phase transformation will be taking place and at a temperature close to body temperature (37°C) GT file will be fully austenitic, while GTX file will be austenite + R-phase.

### 3.3. Finite Element Results

**Figure 5:**
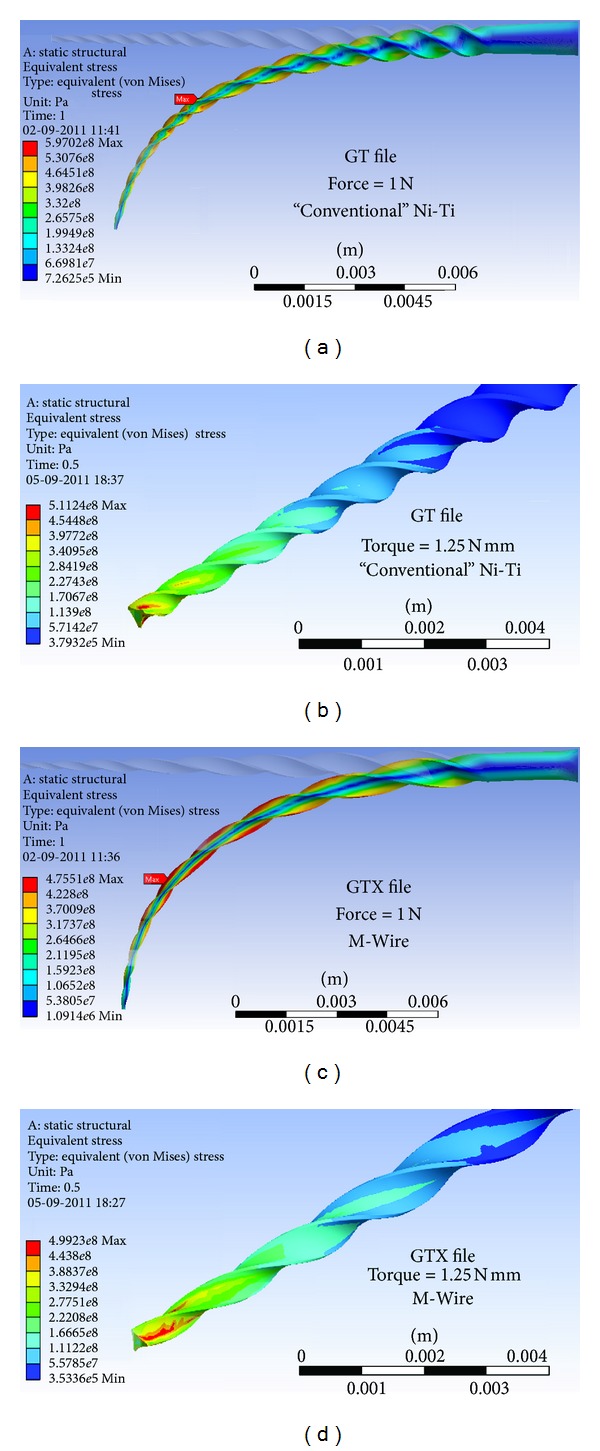
((a), (c)) Flexural von Mises stress distribution for a 1 N load at the tip. ((b), (d)) Torsional von Mises stress distribution for a 1.25 N mm torque with the file clamped 4 mm away from its tip.

**Figure 6:**
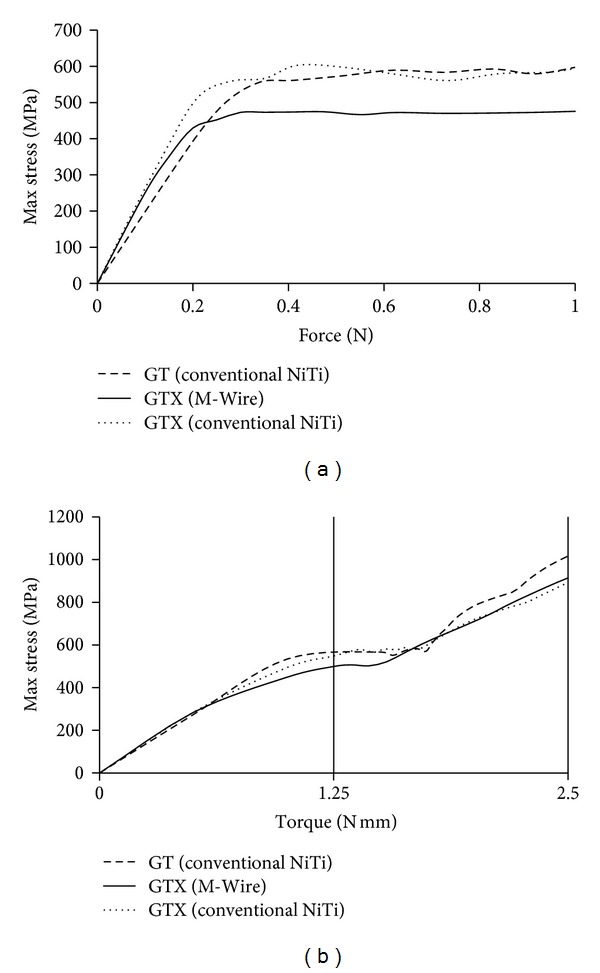
(a) Evolution of the maximum stress for bending simulation (up to 1 N). (b) Evolution of the maximum stress for torsion simulation (up to 2.5 N mm).

Some representative results of the von Mises distribution along the files are shown on Figure 5. The stress *versus *load relationships in both bending and torsion are shown on Figure 6.  Table 1 summarizes the whole results.

## 4. Discussion

### 4.1. X-Ray Diffraction Analysis

There are differences between GT and GTX alloys, namely, different phases present at equal temperatures. The three Ni-Ti alloy phases can be identified through X-Ray Diffraction, although the temperature range used in this study (*T* = [−40°C; 100°C]) is not enough for identifying martensite, which occurs at lower temperatures.

The wide test temperature range was defined not only for encompassing clinically relevant temperatures (near 37°C) and ambient temperature, but also because it is known [31] that there is a phase transformation between *T* = −20°C and *T* = 80°C. Although files are sterilized at temperatures higher than 100°C, there is no need to reach such large values as austenite is known [31] to be the only phase present beyond 100°C.

XRD (and DSC) data show that R-phase appears at higher temperatures for GTX file than for GT file. R-phase is present in GT file's alloy in the range of temperatures close to 10°C in the cooling cycle. On the other hand, GTX file's alloy has R-phase around 40°C both in cooling and heating cycles. This means that, at body temperature (*≈*37°C), GT is austenitic and GTX already presents a significant amount of R-phase. R-phase allows for stress relaxation during adaptation of an endodontic file to root canal curvatures; thus, loads needed to deform it, either in bending or in torsion, are much smaller than those needed to deform austenitic instruments.

R-phase is many times not taken into consideration when designing Ni-Ti alloys for endodontic rotary files, although this crystallographic phase brings important mechanical properties, such as a lower Young's modulus when compared to the austenitic phase. Consequently, its flexibility is larger so the internal stresses of the material inside a given curved root canal are smaller, which increases mechanical resistance under fatigue cyclic loading [29].

In conclusion, XRD analysis showed GTX files to have R-phase at body temperature (37°C) and may, therefore, be better suited for instrumentation of curved canals than GT files (which are austenitic at body temperature).

### 4.2. DSC Analysis

The DSC curves show, during cooling, a clear shift of the transformation temperatures from peak temperature close to 35°C for GTX file to a peak temperature close to 5°C for GT file. A similar shift is also found, during heating. The trends of the curves for both files during cooling let us presume that cooling until −40°C gives rise mostly to A*⇔*R but also a minor amount of martensite may be formed. This analysis is consistent with the XRD data previously presented.

### 4.3. Finite Element Analysis: Geometrical Comparison

As far as a geometrical comparison is concerned, from Table 1 one may consider that the deformation differences between the GT and GTX geometries are residual in bending (4.4% difference when the deflection is measured on the tip for a 1 N force). On the contrary, for the same torque and boundary conditions, the GTX geometry alone is already introducing flexibility with approximately twice the angular deformation of the GT file in both simulations.

It is important to notice that, as the files are subjected to a heterogeneous stress distribution (shown by the colour gradient visible from the central axis to the boundary, both in bending and in torsion), different phases are present throughout the file's cross-sections. That is why a stress relaxation occurs when a certain threshold is achieved (visible on the plateaus in Figure 6) that is due to the material phase transformations happening during the deformation. At these regions the deformation will increase with the load level, but the maximum stress in the material will not suffer a significant change.

### 4.4. Finite Element Analysis: Materials Comparison

A comparison between GT and GTX files was also made taking into account the material model differences. The less stiff M-Wire, with a lower phase transition stress level, considerably increased the flexibility of the GTX file. For the same bending force (1 N at the tip), the GTX file was 27% more flexible than its GT counterpart (7.29 mm tip deflection against 5.98 mm). Likewise, the maximum stress measured in GTX was 19% smaller than the one measured in GT, which may correspond to an enhanced endurance of GTX instruments clinically, although this suggestion deserves further investigation by considering dynamic rotation inside root canals. Also, for the same torque, GTX was considerably more flexible than GT, with a twist practically three times as large. The M-Wire GTX files are showed to be considerably more flexible and capable of stress relief at the most critical zones than GT instruments. Other than temperature effects, this increased flexibility is due to a smaller Young's modulus.

## 5. Conclusions

In conclusion, the relevant combination of FE results with results from DRX and DSC made it possible to conclude that there are flexibility related benefits in M-Wire files at body temperature (37°C). Also, under conditions of the present study, it is suggested that this property might reflect positively on the dynamic rotation inside the root canals which is seen in the clinical situation.

Further study is still required to include the effects of cyclic loading in the performance of the endodontic rotary files. In that case, files should be modelled by using the SMA model instead, which includes hysteresis (different mechanical behaviour during loading and unloading). Nevertheless, extrapolation will still be needed, as this model does not envisage the accumulation of residual stresses or hardening.

## Figures and Tables

**Table 1:** Summary of the Finite Element modelling results (GT file's results were used as reference for the relative deformations and stresses).

	Geometry	Material	Absolute deformation	Relative deformation	Absolute maximum stress	Relative maximum stress
Force 1 N	GT	NiTi	5.73 mm	—	586 MPa	—
	GTX	NiTi	5.98 mm	+4.4%	594 MPa	+1.4%
		M-Wire	7.29 mm	+27.2%	476 MPa	−18.8%
Torque 2.5 N mm	GT	NiTi	53°	—	1016 MPa	—
	GTX	NiTi	109°	+105.7%	893 MPa	−12.1%
		M-Wire	133°	+150.9%	914 MPa	−10.0%
1/2 Torque 1.25 N mm	GT	NiTi	13°	—	511 MPa	—
	GTX	NiTi	23°	+76.9%	548 MPa	+7.2%
		M-Wire	39°	+200.0%	499 MPa	−2.3%
